# Young mothers’ use of and experiences with mental health care services in Ontario, Canada: a qualitative descriptive study

**DOI:** 10.1186/s12905-022-01804-z

**Published:** 2022-06-07

**Authors:** Susan M. Jack, Eric Duku, Heather Whitty, Ryan J. Van Lieshout, Alison Niccols, Katholiki Georgiades, Ellen L. Lipman

**Affiliations:** 1 School of Nursing, McMaster University, HSC 3H48B, 1280 Main St. West, Hamilton, ON L8S 4L8 Canada; 2 Department of Psychiatry and Behavioural Neurosciences, McMaster University, Hamilton, ON Canada; 3 Institute for Innovation and Implementation, School of Social Work, University of Maryland Baltimore, Baltimore, USA; 4 Ron Joyce Children’s Health Centre, Hamilton, ON Canada

**Keywords:** Adolescent, Pregnancy, Postpartum, Mental health disorders, Qualitative research, Health service utilization, Mental health services

## Abstract

**Background:**

Despite the high prevalence of mental health issues among young mothers, their subsequent needs for mental health care support does not correlate with their access and use of services. The purpose of this study, grounded in the experiences of young mothers living in Ontario, Canada, was to describe their experiences of using mental health services during the perinatal period, and to identify the attributes of services and professionals that influenced their decision to engage with mental health services.

**Methods:**

As the qualitative component of a sequential explanatory mixed methods study, the principles of qualitative description informed sampling, data collection, and analysis decisions. In-depth, semi-structured interviews were conducted with a purposeful sample of 29 young mothers (≤ 21 years) who met diagnostic criteria for at least one psychiatric disorder, and who were ≥ 2 months postpartum. Interview data were triangulated with data from ecomaps and a sub-set of demographic data for this purposeful sample from the survey conducted in the quantitative study component. Qualitative data were analyzed using both conventional content analysis and reflexive thematic analysis; the subset of survey data extracted for these 29 participants were analyzed using descriptive statistics.

**Results:**

Young mothers identified the need to have at least one individual, either an informal social support or formal service provider who they could talk to about their mental health. Among participants deciding to seek professional mental health support, their hesitancy to access services was grounded in past negative experiences or fears of being judged, being medicated, not being seen as an active partner in care decisions or experiencing increased child protection involvement. Participants identified organizational and provider attributes of those delivering mental health care that they perceived influenced their use of or engagement with services.

**Conclusion:**

Organizations or health/social care professionals providing mental health services to young pregnant or parenting mothers are recommended to implement trauma-and violence-informed care. This approach prioritizes the emotional and physical safety of individuals within the care environment. Applying this lens in service delivery also aligns with the needs of young mothers, including that they are actively listened to, treated with respect, and genuinely engaged as active partners in making decisions about their care and treatment.

## Introduction

Globally, the proportion of births to adolescent mothers has declined over the last 30 years, at least in part due to a trend of mothers postponing childbearing [1]. Still, between 2016 and 2020, up to 8600 births/year in Canada and 3000 births/year in Ontario were from teen mothers [2], approximately 2% of total births in the country and province. Experiencing pregnancy in adolescence may disrupt developmental and life course trajectories into young adulthood, including limiting opportunities for post-secondary education and employment [3–5]. For many adolescents, the transition to motherhood has “the power to transform teens’ lives in meaningful and positive ways” [5] (p. 7). However, in this population, histories of adverse childhood experiences (ACES) often contribute to teen mothers’ poor educational, employment, socioeconomic, and physical and mental health outcomes in young adulthood [6, 7].

Canadian data suggest that up to 20% of young mothers struggle with postpartum depression [8]. Studies from around the world indicate that up to 44% of adolescent mothers develop postpartum depression [9], twice the rate of older mothers [9, 10] and childless female teenagers [11]. There is less information for other mental health disorders, but data from Brazil [12] and Canada [8] suggest that these are elevated as well, even when structured diagnostic interviews are utilized. Studies from the United States and New Zealand report that more than 30% of young mothers endorse major depressive disorders and anxiety disorders up to 5 years after the births of their infants when similar assessment methods are used [13].

Despite the high prevalence of mental health disorders among teen mothers, their subsequent needs for mental health care does not correlate with their access and use of services [14, 15]. At a systems level, most communities with publicly funded health care systems report insufficient specialized mental health services to meet the needs of pregnant and postpartum youth [16]. This leaves many young mothers to seek out mental health support from primary care providers [17], who report varied levels of knowledge, skills, or confidence to treat the potentially complex mental health needs of this population [14, 18]. Additional barriers to access include limited resources for transportation and childcare, and a reported lack of time related to balancing the competing demands of self-care with parenting and education or employment [14, 15, 19]. Young mothers also report that the stigma associated with needing mental health care, along with a fear of caregivers learning that they are seeking these services, pose additional barriers [14, 15].

### Young Mothers Health study

The Young Mothers Health Study was conducted to measure the rates, types, and severity of mental health disorders among young mothers (defined as individuals 15–20 years at time of birth of first-live infant) living in Ontario, Canada. A secondary objective was to describe their use of health and mental health services. In this sequential explanatory mixed methods study, quantitative and qualitative data were collected and analyzed in two consecutive phases. In the first phase (2012–2015), a representative cohort of 450 young mothers were recruited who met the study inclusion criteria: (1) female ≤ 21 years old; (2) pregnant or parenting a child < 2 years; 3) able to speak and understand English; and 4) living in Hamilton, Ontario, Canada. Data on 100 older comparison mothers were also collected. Participants were recruited through social media and targeted advertisements at community agencies, including residential maternity homes, public health home visiting programs, primary care clinics, and through prenatal care providers. Each participant completed a demographic questionnaire, the MINI-KID structured diagnostic interview [20] to collect DSM-IV psychiatric diagnoses (lifetime or current), the Edinburgh Postpartum Depression scale [21], and questions adapted from the Canadian Community Health Survey [22] about their health service utilization, including type of practitioner and frequency of usage. Among this sample, approximately two-thirds (64%) of young mothers reported a mental disorder, with 37% reporting more than one disorder [8]. A detailed description of these quantitative methods and findings are published elsewhere [8]. In this article, we report on the methods and findings from the descriptive qualitative study that was conducted in the second phase (2014–2016). The objectives of the qualitative study component were to describe:

1. The types of support and services used by young mothers;
2. Factors that influenced young mothers’ decisions to seek mental health care;
3. Young mothers’ perceptions regarding the attributes of services and professionals that influence their engagement with services; and
4. Their recommendations on how mental health care professionals can better engage with this population of clients.

## Methods

The principles of qualitative description informed sampling, data collection, and analysis decisions in this applied qualitative health research study [23]. As a naturalistic inquiry process, this type of qualitative design allows for the rich description of the phenomenon of interest under study, informed by the experiences of the participants, and to be reported by staying close to participants’ words. Methodologically, qualitative descriptive studies typically employ non-probability convenience or purposeful samples. In-depth interviews are generally a primary data collection strategy to document participants’ descriptions and experiences of events. Varied approaches to content or thematic analysis are then employed to code and interpret the data [23]. This approach is commonly used to guide the qualitative component of mixed methods studies [24].

### Sample

From participants who completed the quantitative survey in the first phase of this mixed methods study and consented to follow-up, a purposeful sample of 29 young mothers who met diagnostic criteria on the MINI-KID for at least one psychiatric disorder, and who were ≥ 2 months postpartum were invited to participate in this qualitative study. Within this purposeful sample, using health service utilization data collected during the quantitative phase, we sought to include an equal number of participants who reported either high or low use of mental health services. Utilization of mental health services was defined as the total number of professional visits in the last 12 months for help with emotions, mental health, or alcohol or drugs. Low service utilization was the lowest quartile of visits endorsed (≤ 2 visits in the last 12 months) and high utilization was highest quartile of visits endorsed (28–74 visits in the last 12 months).

### Data collection

For these 29 participants, descriptive data from the questionnaire completed in the first quantitative phase of this mixed methods study were utilized. Data to describe their demographic characteristics, descriptions of the rates and types of mental health disorders, and type of providers accessed were extracted. In the original questionnaire, each participant completed the MINI-KID structured diagnostic interview; these data were extracted to report on the type and rates of mental health disorders.

Each participant was then invited to participate in a single, in-depth semi-structured interview to learn about their experiences of parenting with a mental health disorder, to understand their needs and preferences for support, and their experiences of that care. Each participant was also asked to provide recommendations on how professionals could best provide mental health services to young mothers. Interviews were scheduled at a time and place that was mutually convenient for the interviewer and participant. Each interview lasted between 30 and 90 min. All interviews were audio-recorded.

At the beginning of each interview, participants were asked to develop an ecomap to visually illustrate the number and types of informal support and formal health and social care services accessed. Participants were also asked to illustrate and describe the quality of their relationships with each source of support. As the ecomap was being developed and informal or formal sources of support were identified, the interviewer asked probing questions to better understand the participant’s experiences, and level of use of each support. Ecomaps have been used successfully as a mode of data collection in research to document personal and family social relationships across a range of vulnerable populations [25].

### Data analysis

The subset of survey data extracted for these 29 participants were analyzed using descriptive statistics. Specifically, frequencies and percentages were used to describe categorical survey data and for continuous data, means and standard deviations were used.

All audio recordings were securely sent to a professional transcriptionist and then transcribed verbatim with identifying information removed. Conventional content analysis [26] of the transcripts was used to identify, code, and categorize the organizational and provider level attributes that participants perceived facilitated (or served as barriers to) their use of or engagement with health care services. Finally, reflexive thematic analysis [27] was employed to construct a rich, comprehensive narrative of participants’ needs for support, experiences of accessing professionals support, and their recommendations for mental health professionals who provide care or services to young mothers. This latter analysis involved a six-phase process completed by the lead author (SMJ) along with reflexive consultations with the senior author (EL) that included: (1) immersion in the data by reading and re-reading the transcripts, making notes to reflect initial observations of what was happening in the data and highlighting meaningful quotes; (2) generating lists of both descriptive and interpretive codes; (3) actively searching for patterned responses and meaning across transcripts in response to the research objectives; (4) reviewing, defining, and naming themes; and (5) organizing the themes to generate a comprehensive and thoughtful narrative of the participants’ experiences [27]. In steps 2 and 3, a four-level social-ecological model was used to generate descriptive codes and categorize factors influencing service utilization [28].

Ecomap data were analyzed by counting and categorizing the different types of formal and informal supports reported on and accessed by each participant within an Excel file. For each support listed on an ecomap, a research assistant then read the corresponding transcript to assign a subjective rating (positive, negative, or neutral) to describe the quality of the relationship between the participant and that specific source of support. This process was duplicated by the lead qualitative analyst (SMJ) to verify the assigned ratings.

All study procedures were approved by the Hamilton Integrated Research Ethics Board, Faculty of Health Sciences, McMaster University (REB #10–585). Participants reported on in this paper provided informed consent to participate in both the quantitative and qualitative study components. All methods were performed in accordance with the relevant guidelines and regulations of the institutional research ethics board.

## Results

### Demographics

A purposeful sample of 29 young mothers (mean age = 18.83) discussed their experiences of seeking social support and health care services for their mental health disorders during pregnancy and the early years of their children’s lives. The sociodemographic characteristics of these young mothers are summarized in Table 1. The majority of the young women identified their relationship status as single (58.6%) with only 37.9% married or in a common-law relationship.

**Table 1:** Sociodemographic characteristics of study participants (*n* = 29)

Sociodemographic variable	*M* (SD)/%
Average age (years)	18.83 (1.20) (min–max: 17–21)
Average number of children	1.14 (0.35)
Born in Canada	86.2
*Highest grade of elementary or high school completed*	
Grade 10 or lower	27.5
Grade 11	41.4
Grade 12	31.1
*Relationship status*	
Married/common law	37.9
Single	58.6
Divorced/separated	3.5
*Living arrangement*	
Live alone (without child)	3.4
Live with child only	31.3
Live with child + partner	17.2
Live with child + partner + extended family	24.1
Live with child + extended family	17.2
Live with child + non-family members	6.8
*Socioeconomic status*	
Currently employed	
Yes	17.2
No	82.8
“Is money a struggle for you?”	
Yes	51.7
No	48.3

### Prevalence and type of mental health disorders

Within these social contexts, the majority of participants (69%) reported parenting with a current mental health disorder at the time of data collection, with 37.9% of the sample reporting a diagnosis of more than one disorder. The average number of disorders across the sample was 1.62 (SD = 1.76, minimum = 0, maximum = 7). Prevalence rates of specific mental health disorders experienced by women in this sample are summarized in Table 2.

**Table 2:** Prevalence rates of mental health disorders among study participants (*n* = 29)

Mental health disorder	%
Major depressive disorder	27.6
General anxiety disorder	10.3
Separation anxiety disorder	13.8
Social phobia	27.6
Specific phobia	37.9
Attention deficit hyperactivity disorder (ADHD)	13.8
Oppositional defiant disorder	20.7
Conduct disorder	10.3
Comorbidity (> 1 disorder)	37.9
Any current disorder	69.0

### Social factors influencing mental health and well-being

As these mothers completed the ecomaps, their narratives commonly included references to ACES including physical or sexual abuse, exposure to intimate partner violence or traumatic losses such as the incarceration, murder, or death of a parent. All of the women were living within conditions of economic and social disadvantage, with many describing life histories complicated by multiple family moves, disruptions in their education, precarious employment and poverty. A common stressor for participants was the responsibility of parenting alone and managing the demands of independent living. A young mother of a single infant shared,

I just moved. So those emotions are getting to me. This is my first time moving out from my mom’s or my dad’s [places]. So, it’s like, “I’m by myself, I have my baby here, but I’m by myself. I have no parents to help me. I have to pay my own rent; I have to pay my own bills. So, it scares me sometimes, and my thoughts, they just sit there. They’re like, “Hey, we’re going to make you cry today.” But ten minutes later, we’re good. [Participant 14, 20 years]

Overall, through triangulation of survey, interview, and ecomap data, it was identified that all participants had histories of trauma, were parenting while experiencing multiple social and economic stressors and managing at least one mental health disorder. Parenting within this context was perceived to negatively impact their overall well-being and increased their needs for mental health services.

### Need for consistent and accessible support

The fundamental need for young mothers navigating mental health disorders while parenting was to have at least one individual in their life who was “always there” to talk to and who was trustworthy, easily accessible, and skilled in active listening. This individual could be either an informal social support (e.g., parent, friend) or a formal professional support (e.g., health care provider, counsellor). What was critical was that the source of support was available to provide help when requested and had the resources, empathy, experience, or knowledge to meet their identified needs. As one mother explained,

What do young mothers need in terms of support? Help when they need it. Especially support in getting through some of the hard times… Having someone close to you, that you can depend on in the first while [after having a baby] and who can give you a bit of a break. [Participant 15, 19 years]

Partners, friends, or family members often fulfilled the role of providing emotional and instrumental support, as well as guidance on parenting and managing stress. Mental health support networks also included engagement with heath care or social services providers. The types of informal social or formal professional supports needed and accessed varied across study participants. Overall, participants experiencing high levels of support identified connecting regularly with at least one supportive friend or family member and engaging consistently with a supportive primary health or social services provider. Having timely access to specialized mental health services to address both emergent mental health crises and long-term conditions was also important to this group.

### Social support networks

Analysis of the ecomap data indicated that participants listed an average of 8 (range 3–16) social supports during their pregnancy and an average of 9 (range 4–18) in the postpartum period. Among their available supports, all participants identified having at least one closely connected and positive relationship with a friend, family member, or partner who provided them with multiple forms of social support during the prenatal and postpartum periods. Yet, most were simultaneously navigating other relationships that were difficult or tumultuous. These stressful relationships were often described as unsupportive and reflected lives with abusive partners or former partners or their own parents’ physical or emotional unavailability. The theme of loss was common across the interviews and ecomaps, with many participants discussing the loss of social networks once they became pregnant, estrangement from extended family, or parental death including by suicide.

### Formal professional supports

The young mothers in this study were well connected to a range of professionals or community service agencies. Almost all participants (97%) engaged with at least one health care provider (e.g., obstetrician, family physician, midwife, or public health nurse) to receive prenatal health care. A majority of participants (72%) also lived in or accessed services from a community-based maternity home that provided residential, health, and education services to young pregnant or parenting girls. Analysis of the ecomap and interview data revealed that most mothers identified using multiple services, with 59% of participants reporting accessing three or more professional supports during pregnancy and 83% accessing professional supports in the postpartum period (Table 3).

**Table 3:** Number of professional supports accessed by study participants during pregnancy or postpartum period as disclosed in ecomaps and qualitative interviews

Number of professional supports accessed	0	1–2	> 3
During pregnancy (% of sample)	3	38	59
During the postpartum period (% of sample)	10	7	83

From the survey data, participants reported completing an average of 16.38 (SD = 19.91; minimum = 0, maximum = 74) professional visits specifically for addressing mental health disorders over the last 12 months. On average 2.14 (SD = 1.53, minimum = 0, maximum = 4) professionals were seen to address participants’ mental health disorders and the types of providers this sub-sample of participants saw or talked to on the phone about emotional or mental health problems or the use of alcohol or drugs in the last 12 months are summarized in Table 4. The most common providers accessed were social workers, counsellors or psychotherapists (58.6%), family doctors/general practitioners (48.3%) and nurses (44.8%).

**Table 4:** Types of health care or social service providers accessed for mental health problems

Type of provider	%
Psychiatrist	20.7
Family doctor/general practitioner	48.3
Another medical doctor (e.g., cardiologist, gynecologist)	27.6
Psychologist	3.4
Nurse	44.8
Social worker, counsellor, or psychotherapist	58.6
Religious advisor such as priest, chaplain, or rabbi	3.4
Midwife	6.9
No one	20.7

### Young mothers’ decisions to seek mental health care

In reflecting on their mental health disorders and support needs, the mothers shared insights as to why they decided to manage their mental health disorders either independently, with the support of family or friends or through seeking professional health or social care services. Participants who chose to manage their mental health independently often saw themselves as needing to be self-reliant. As a 21-year-old mother of one child explained, “I always feel like I can deal with things myself. It would be a good idea if I did (seek professional support), but I probably won’t, just because I’m more of a strong-headed, stubborn person” [Participant 20]. Their reasons for not seeking support however were underpinned with language that signaled feelings of vulnerability, fear, or worry about discussing their mental health with a professional. For some, their words alluded to concerns that if they could not help themselves, no one could. As one mother shared,

If you can’t handle [your mental health issues] by yourself, how do you expect someone else, who doesn’t even know you, to do it? Like, if I can’t handle my anger, I can only imagine how I’m going to freak out when someone else is trying to tell me how to control it right? I don’t like being told what to do. [Participant 18, 18 years]

An understanding of their health history, accessibility, and a sense of trust and connection were rationale provided for connecting with families or close friends for help in managing mental health disorders. One mother, who had a very positive relationship with her parents and siblings, expressed that:

I feel like I can relate more to my family and they can relate to me more; so, they can help me with an issue. So, if I’m feeling upset, my mom or my sisters tell me, “That’s normal. That’s not normal. You’re fine. It’ll pass. Or whatever.” I don’t know if that’s different or not, but a professional who would be helping me from an agency would be doing the same thing. So, I just feel more comfort knowing that my family is there. And comfort is the biggest thing. [Participant 20, 21 years]

Among those deciding to seek professional help, many of their narratives were underpinned however with a hesitancy to access mental health care. This hesitancy to seek help was often related to a fear of being judged by others and an acknowledgement of the stigma attached to experiencing mental illness and being a young mother. There were also fears about perceived negative outcomes they associated with accessing mental health care, such as being medicated or being reported to child protective services. For many participants, their hesitancy to seek care was also grounded in past negative care experiences. For other participants, there was an acute awareness that the symptoms associated with their mental health disorder significantly influenced their behaviours and motivation to be able to locate, access, and then participate in mental health services or treatments.

For those who ultimately sought professional care, many referenced an increased openness and willingness to seek and accept professional support during their pregnancy. The transition to motherhood was also commonly identified as a primary motivating factor to seek mental health care. One mother [Participant 26, 21 years], with two young children stated, “it’s not just me anymore. It (seeking mental health care) was for the baby.” Another mother shared that making this decision also required the confidence to dismiss worries about stigma and others’ perceptions of her decision to seek professional help:

It’s not about them. It’s about you and you do what you have to do for you and your child. So, don’t do what’s better for everyone else. I didn’t care what everybody else thought because I was doing it for me, I wasn’t doing it for them. Whatever I did was just to help me out and to make things easier on me. [Participant 15, 19 years]

Past experiences with health care professionals also influenced their decisions to seek mental health care in pregnancy or the postpartum period. Young mothers who shared examples of positive health care experiences during the prenatal period were more confident in their decision to accept a referral for mental health care. These positive past experiences were illustrated with examples of health care professionals who provided reassurances, identified and acknowledged the young mothers’ strengths, explained procedures, offered help, and outlined options for care, thus allowing the young mother to have control over her decisions.

In comparison, the mothers who shared their stories of negative and traumatic experiences with health care professionals, including during the prenatal and intrapartum periods, were much more fearful and hesitant to re-engage with the health care system to address their mental health disorders. In particular, their narratives included exemplars of not being listened to nor having their experiences validated, their requests for information or help being ignored, and the use of power by health care professionals to override their decisions or requests for services.

### Factors influencing young mothers’ use of mental health care services

Participants discussed multiple factors (Table 5) that served as either facilitators or barriers in their decision-making to use mental health care services.

**Table 5:** Factors influencing young mothers’ use of mental health care services

Facilitators	Barriers
*Social factors*	
	Fear of negative consequences associated with seeking mental health care, including potential report to child protective services Perceived stigma of accessing mental health services and being a young mother
*Organization or community attributes*	
Appointment management Option to book, cancel, or re-schedule appointment via “text” (SMS) Service coordination Co-location of health, education, social services Access to services or providers Primary care provider model Options for home visits available Services or provider available to respond to acute mental health needs Voluntary participation	Service environment Decreased sense of privacy with presence of cameras Not easily accessible via public transportation or to navigate with infant/stroller Appointment management Punitive response to cancelled or missed appointments Limited options for contacting organization Access to services or providers Mandatory participation Limited access to affordable mental health care Service coordination Lack of care coordination or information sharing between providers, resulting in requirement to repeat social and mental health history to multiple providers
*Provider attributes within the context of a therapeutic relationship*	
Establishment of good client-provider therapeutic relationship Demonstrates care and understanding when appointments need to be cancelled or re-scheduled Demonstrates genuine concern and sincerity Is respectful and non-judgmental Prioritizes client needs or concerns Respects client’s time and decisions Fully present during encounter by limiting distractions (i.e., forms, phone) and does not appear rushed Communication skills Skilled active listener Provides anticipatory guidance Validates client’s experiences Able to clearly communicate information about client’s mental health disorder Allows client to control narrative; does not pressure client to talk or disclose information before they are ready to Planning and delivery of care Identifies and prioritizes client needs or concerns Engages in shared decision-making, providing client with choice and control over final decisions Offers options for care or service, provides detailed description of each option Assesses client’s understanding of each option Co-develops plan of care with client that is perceived as helpful to address priority needs and feasible to implement Creates time and space to assess client’s perceptions and expectations with respect to treatment/services Seeks client’s permission or consent to share information with other professionals or to allow other individuals to be present during an appointment Actively assist client in making, confirming and accessing referrals, including identifying and addressing any barriers	Unsafe client-provider relations Provider perceived to be punitive when client misses or cancels appointment Does not prioritize client privacy or emotional safety Infantilizes client by speaking to other adults (partner/family member) instead of client, speaks in a condescending manner Perceived to be overly intrusive and judgmental Planning and delivery of care Provides advice or directly tells client what to do Dismisses client’s concerns, needs, or experiences
*Individual attributes*	
Help-seeking behaviours Social or family supports met identified needs Expressed preference for informal support over formal supports Past positive experiences with health care providers	Help-seeking behaviours Preference to manage stressors/mental health disorders independently Past negative experiences with health care providers or social services

### Barriers to using mental health care services

There was consensus that managing logistics around getting to community-based agencies that provide mental health services was a primary barrier. Participants confirmed that most, if not all, agencies provided bus tickets or taxi coupons to help the mother attend her appointment. While this small financial support was perceived to be necessary and important, it was evident that getting to the appointment was actually a much more complex, and physically and emotionally demanding process for young mothers with infants. They described complex lives where they are responsible for the health, safety, and well-being of themselves and their infants. Within this context, they manage, juggle, and coordinate appointments with multiple programs, services and professionals and do so in the absence of private transportation and financial resources. The participants emphasized that this work of care coordination was time-consuming and exhausting. At times, it even meant not being able to attend an appointment or program, as one participant explained:

I didn’t really have time to do the program because I was pretty much a single mom, doing it all on my own. So, it was hard, like, it wasn’t too hard to get to the programs, it was just to find time to do them and trying to catch up on sleep and get everything else done.” [Participant 1, 20 years]

For other mothers, the complexity and challenges were found within the action of getting to the program or appointment. Arriving on-time to an appointment often required planning around the infant’s schedule, for example,

After you have the baby, it’s difficult to get to an appointment. It’s more difficult because you have to worry about, “Oh is my child going to be hungry during this time? What do I need to pack? What if the bus runs late? Am I going to miss my appointment?” [Participant 3, 21 years]

The young mothers revealed that navigating small physical spaces, including on public transportation, and accessing buildings with a stroller, often in inclement weather without anyone else to help, and exposed to the judgments and “rude comments” of strangers was exhausting.

For young mothers with anxiety disorders, navigating the logistics of finding an agency for the first time and not knowing “what to expect” at the appointment were highlighted as additional stressors. Returning home, again often on public transportation, was difficult for some mothers who spoke about not having a private space to reflect on what happened within the client-clinician encounter or to express post-appointment emotions.

Organizational policies that reduced flexibility or limited communication to book or re-schedule appointments were barriers to consistent engagement with services. A hesitancy to return to service organizations also occurred when participants experienced a lack of privacy or a threat to their emotional safety through multiple requests to repeat their medical histories.

### Facilitators to accessing mental health care services

Based on their varied experiences of engaging in a wide range of mental health-focused services, participants were able to describe specific attributes of organizations that facilitated their engagement with mental health services. There was a clear preference for working with organizations where health and social services were co-located. Access to a consistent or primary care provider, and the option to receive services through home visits were also highly valued attributes. One mother, with two young children, explained,

Having a public health nurse come to your house is a huge advantage. There are tons of emotional advantages, tons of intellectual advantages. It was a huge thing for me and it’s easier with a newborn to have the nurse come to me and to work around my schedule. [Participant 26, 21 years]

An organization’s flexibility and responsiveness in scheduling an appointment at a time convenient for the mother, or “fitting her in,” so that an imminent mental health concern could be addressed in a timely fashion was highly appreciated. As one mother explained,

My doctor’s office is very flexible. If you are like, “Oh, look, like this is important, I really need to get in,” they put you in the earliest time they can which is great. They always answer their phones. [Participant 3, 21 years]

The use of cell phones was a constant in these mothers’ lives, and they identified that being able to text the organization or provider to book, cancel, or re-schedule an appointment facilitated better communication. Finally, some participants indicated a higher degree of engagement with voluntary programs, in comparison to a program that they were mandated to attend.

### Provider attributes that influence engagement with mental health care services

Reflecting on their experiences with multiple providers, participants provided a consistent list of provider attributes that were highly valued. The mothers were most likely to engage and consistently work with providers with excellent communication skills, the ability to develop a therapeutic relationship characterized by trust, and who prioritized exploring and understanding the client’s goals of care.

The opportunity to access care or support at the time they were experiencing a high number of stressors or mental health issues was of primary importance. In parallel, because of unpredictable or changing events in their lives, participants also appreciated working with providers who had the flexibility to re-schedule an appointment at short notice and who demonstrated understanding about the mother’s need to change or cancel an appointment. Being able to communicate via text also facilitated last minute changes. One young mother, in speaking about her nurse home visitor explained,

And she [the public health nurse] texts if we have an appointment. Like it’s perfect. And then if I have to say, ‘Oh, there’s an emergency, can I see you sooner?’ Then she’s like, ‘Yeah, of course. These are the days I have free…. And when are you available?’ And then she comes to me. [Participant 3, 21 years]

To be able to discuss their mental health concerns it was paramount to the young mothers that they feel “connected” to their provider. With respect to the therapeutic relationship, the mothers sought out genuine connections and appreciated when their need to seek support was valued and not perceived as a burden. As one mother summarized,

I connected with her [the addiction counsellor] so good because I felt that she was so down to earth, and I could open up to her. So, I opened up and I told her everything. I guess she then switched to work at another site and so a new counsellor came in. I was actually able to open up to her too, because she also worked shifts at [the maternity home]. So, I was like, “Okay, you know, I’ve met her before. She’s cool. I’ve talked to her.” And now I can open up to her with anything. [Participant 12, 19 years]

Within the care encounter, participants expressed a preference to work with providers who were respectful and non-judgmental and who created a safe space where they could speak privately and in confidence. There was an appreciation for providers who, regardless of the mother’s early resistance to engage, recognized that the process of building trust takes time and stayed committed to the process. One mother explained,

I didn’t get along with [my counsellor] at first. When I started going there, I was a rebel, I didn’t like them, and I use to tell them off. But they were there for me. So, I just started liking them more. Eventually, I was just like, “Okay, you guys are actually here to help me. Like, not everybody is out to get me.” So, I don’t know…I just built a bond with them and I still have it now. [Participant 4, 21 years]

Repeatedly, mothers expressed appreciation for professionals who were skilled active listeners. Equally important was to work with a professional who prioritized identifying the mother’s most immediate needs, and then instead of focusing on the professional’s “agenda,” explored strategies to address priority concerns. When a referral to another clinician or agency was needed, participants valued when their primary mental health care clinician would explain the different options for the referral, outline in detail what services would be offered, and then actively assist in connecting the participant to the next agency.

In contrast, participants identified the negative attributes of client-provider relationships that limited their use mental health services. When participants experienced what they perceived to be a lack of physical privacy or confidentiality, then they were hesitant to discuss their mental health. For several participants, they found it particularly upsetting to be asked about sensitive and personal experiences when other family members were present. The participants did not value working with providers who controlled the agenda, did not listen to their description of their immediate needs, or who made assumptions about their needs and told them what to do without exploring the acceptability of those options to them. When participants perceived that the service provider was judging their behaviour or parenting, they were reticent to continue to seek services. Several participants identified that providers’ questions and words were experienced as intrusive and made them feel guilty or overwhelmed. One mother [Participant 12, 19 years] explained, “Having to manage and navigate a thousand and one questions like ‘Why didn’t you come sooner, blah, blah, blah…it’s too much.” To avoid these experiences and feelings, it was easier to no longer continue with the provider.

### Young mothers’ recommendations to mental health care professionals

Within the interviews, the participants were asked to reflect on their experiences and share recommendations on how mental health care professionals could better engage and support young mothers. The key recommendations are summarized, staying close to the participants’ own words, in Table 6. It was important to participants that professionals providing specialized mental health care recognize and address the social and economic stressors impacting the mothers’ mental health in addition to treating their mental health disorders. The remaining recommendations reflect the young mothers’ needs to be able to engage in a therapeutic alliance where they listened to, respected, and actively engaged in decision-making.

**Table 6:** Young mothers’ recommendations for mental health care professionals

Recommendation	Strategies to achieve the recommendation
1. Understand the connection between mental health and social and economic circumstances	For young mothers, often parenting alone, mental health is connected to their social and economic circumstances. Be prepared to understand and address not only mental health concerns, but social determinants of health including how to access affordable, safe housing, income support, safe childcare and employment opportunities
2. Be present and personable	In each encounter, take a few minutes to get to know the client as an individual. Be warm, genuine and interested in their responses. Being present means not looking at the phone, multi-tasking, looking “rushed” or trying to quickly get through the appointment
3. Be respectful	Young mothers want to be understood and respected. Avoid treating them like a child which means NOT talking down to them, telling them what to do, talking to their parents instead or sharing their information/experiences with family members/other services without consent. Do not rush to judge their language or behaviours without taking the time to understand their circumstances and identify their strengths
4. Be patient	Recognize that it may take time for a young mother to feel safe to share her experiences, to be able to even describe what she is feeling, or to articulate what she needs. Be patient and don’t rush the session
5. Actively listen	Ask questions and listen without interruption. It may take time for the client to feel safe enough to share their experiences, so go slow, ask general questions first and listen to what they share
6. Minimize the use of medical jargon	Recognize that young mothers may not speak or understand the language or terms used by health care professionals. The burden should not be on the client to try to figure out or interpret the information being shared with them. Avoid using medical jargon; when a medical or health term is used (e.g., even a diagnosis of depression or anxiety, or a referral to “CBT”), pause and ask the client to share their understanding of the term, ask if they have questions, and provide clarification respectfully as required
7. Engage in agenda-matching	There is often a mismatch between what the client needs immediate support with and what the provider wants to first address. Prioritize the client’s needs; ask them to identify issue that is most important for them to resolve first. Sessions should be client-centered and not driven by the provider’s “agenda.”
8. Explore options and offer clients choices	When determining a next course of action, treatment, or referral to another service, share the range of options with the client. Explain and describe each option. Avoid the use of the term “mandatory” or any language that infers to the client that they have “no choice” and that they “must” complete a program (e.g., complete an anger management program)
9. Focus on solutions	While it is important for clients to understand their mental health issues, they would like to be able to leave each session with practical solutions and strategies, tailored to their circumstances, that they can implement to “move forward.”

## Discussion

In this qualitative study, we describe the experiences of accessing and using mental health supports from the perspectives of a Canadian sample of young mothers with a mental health disorder. Prior to focusing their discussions on their experiences with specialized mental health services, the young mothers shared detailed narratives of the complex conditions in which they parented, often grounded in histories of loss, trauma, and other ACES. Among young women, the graded relationship between ACES, and in particular experiences of cumulative trauma, and adolescent pregnancy has been well documented [29–31]. The participants described managing the transition to parenthood, most frequently as a lone parent, within conditions of deep social and economic disadvantage while also navigating the well-documented social stigmatization of being a teen mom [5, 15]. Parenting within these conditions have been identified as stressors that contribute to their overall experiences of poor mental and physical health [32]. Not surprisingly, the range of physical health, mental health, and social services they described using reflected their needs to access resources related to housing, education, and parenting as well as services to address their physical and mental health needs.

In response to addressing both their stressors and mental health disorders, all participants identified having a mix of both social support from family, friends, or a partner as well as a therapeutic relationship with at least one professional support through pregnancy and into the postpartum period. While the number, types, and quality of support varied across participants, the one consistent finding was that young mothers needed at least one individual (informal support or professional) who they could talk to about mental health at a time that is convenient for them and when their need is greatest. In a systematic review and meta-ethnography of young mothers’ perceptions of their mental health and well-being in the perinatal period, the presence of a circle of social support was identified as critical for navigating a range of stressors [32]. Social and family supports were found to have a positive influence on maternal mental health by providing instrumental support to reduce parenting stress, validating their experiences, and encouraging the young mother to seek professional mental health care as needed [32]. However, while social support can serve as a protective factor, young mothers in our study also had relationships with family or partners that were tenuous and sources of tension and conflict. The absence of a strong social support network then speaks to the importance of having a wide range of community-based mental health services accessible to young mothers.

Our findings reflect and confirm that multiple system, organizational, provider and client factors influence adolescents’ attitudes and behaviours to seeking or using mental health services [14, 33–35]. While access to and availability of affordable mental health services in high-income countries are oft-cited barriers limiting adolescents’ engagement with services [36, 37], among our purposeful sample of urban Canadian mothers, there was a high degree of knowledge about and experiences with a broad range of community-based and specialized mental health services. Similarly, transportation to appointments is regularly identified by this population as a common barrier to service access [35, 37]. Yet our findings deepen our understanding of this issue from a young mother’s perspective. The barrier reflects not only the ability to access or afford transportation but the complexity of the logistics of travelling to an appointment while navigating public spaces with an infant. The young mothers also discussed how difficult it was to return home via public transportation after an appointment and not having a private space to process their emotions; making them feel even more vulnerable to judgment from others. Stigma around seeking mental health services remains a profoundly common barrier to care among this population [14, 35, 38]. Perceptions of judgment also extend to their interactions with service providers, specifically with respect to their capacity to parent while coping with a mental health disorder [32]. Young mothers in this study were acutely sensitized to the risk that increased engagement with the health care system to address their mental health needs may result in a report to child protection services. This awareness of the surveillance role assumed by many service providers further influences if and how young mothers engage with services or programs offering mental health support [39].

Despite these barriers, the majority of participants had accessed and were receiving mental health support starting in pregnancy and extending into the postpartum period, with services most commonly being provided by social workers, family physicians or nurses. There is substantive evidence that when non-specialist providers are able to offer evidence-informed counseling interventions, that this has the potential to lower depression and anxiety scores in the perinatal period [40]. Thus, this speaks to the importance therefore of amplifying the capacity of primary and public health care systems to identify, assess, and address common mental health issues when and where young mothers need them, including through home visit or telehealth options. This recommendation aligns with the Mental Health Commission of Canada’s Youth Council’s priorities for enhancing the primary health care role in mental health [41]. The challenge though becomes one of supporting primary health care and public health services to increase opportunities to identify and respond to mental health concerns among existing client rosters as well as to provide training to increase health care professionals’ knowledge, skills, and confidence to apply appropriate evidence-based interventions to address client needs.

Co-locating mental health counsellors to provide specialized mental health care within these settings would facilitate client access to these types of supports. From a public health perspective, the implementation of nurse home visiting programs specifically tailored to meet the needs of girls or young women  is one solution to providing support to address a broad range of health and social needs among this population. The Nurse-Family Partnership (NFP) program is one example of an early intervention home visitation program where nurses start visiting early in pregnancy to address the needs of young, first-time mothers experiencing social and economic disadvantage [42]. To address nurses’ requests for additional education and resources, to screen for, and then respond, to maternal depression and anxiety, the NFP has invested resources in developing, evaluating, and mobilizing new mental health innovations within this program [43]. Within universal public health home visitation programs, there are also opportunities to train public health nurses to provide psychotherapy to new mothers experiencing postpartum mood disorders. There is evidence that despite not having extensive previous psychiatric training, public health nurses may be able to effectively deliver evidence-based psychotherapies to this population [44, 45].

However, the deeper purpose of this study, was to understand from the young mothers’ perspectives, grounded in their experiences as health service users, what conditions influence their decisions to seek or engage with existing mental health services. A perceived lack of emotional or physical safety within care contexts, at either the organization or provider level, was a prevailing theme across their narratives. The quality of the client-provider relationship has been identified as an important factor influencing young adults’ intentions and actions to seek support for their mental health [36, 37]. A response to this problem is grounded in the mothers’ expressed needs, preferences and recommendations for mental health services and providers to attend to their histories and social realities, prioritize their safety, provide opportunities for shared decision-making, and recognize and build upon the mother’s strengths. These findings, derived through an inductive process of analysis, mirror the principles of trauma-and violence-informed care (TVIC).

There are four, inter-related TVIC principles that can be applied to organizational policies and procedures as well as within individual provider-client interactions: (1) understand trauma, violence and its impacts on people’s lives and behaviour; (2) create emotionally and physically safe environments for all clients and providers; (3) foster opportunities for choice, collaboration, and connection, and (4) use a strengths-based and capacity-building approach to support clients [46]. If the goal is to create teen-friendly mental health services, where young mothers feel confident in engaging in an ongoing, trusting therapeutic relationship with a service provider, then the solution may be nested in implementing TVIC within specialized mental health care services as well as in any agency/service that provides professional services to this population. This approach to service delivery and care expands on the established principles of trauma-informed care [47], which focus on creating safe spaces that reduce the potential for future harm for clients assumed to have trauma histories, by also accounting for the intersecting impacts of structural, institutional, and social conditions that perpetuate harm [48].

In applying this lens, a critical first step is for youth-serving organizations and providers to cease the labelling of young mothers as difficult to engage [49]. This term places blame on the individual; instead providers should re-frame their understanding of the issue to one of recognizing that the problems of hesitancy and reduced use result from services being difficult to access and at times, unsafe, to engage with. Creating opportunities to provide TVIC training to staff and providers [50] will create opportunities for organizations to become “trauma-and violence-informed” and rather than problematizing adolescent behaviours, support staff to develop a deeper understanding that young mothers’ histories of trauma and experiences navigating systems that perpetuate harm are often at the root of behaviours that limit further engagement with health care and social service organizations.

Then, any organization or clinical service providing care to young mothers might consider reviewing scheduling, communication, and service delivery models. When possible, providing flexible options for scheduling and booking appointments at a time convenient for the client, as well as increasing staff and providers’ understanding of why young mothers may cancel appointments at short notice given competing demands, would be valued. Offering walk-in, in addition to scheduled, appointments may also improve access [51]. Given the near ubiquitous ownership of cellphones [52] and preferences for short message services (SMS; or “texting”) by adolescents [53], providing texting options to communicate and schedule appointments might further facilitate access. Furthermore, there is emerging evidence that providing tailored mental health text messages or services may be a safe, feasible, well-accepted strategy to provide adjunct support to young mothers experiencing postpartum depression [54]. As we move towards the post-COVID-19 pandemic period, this will create opportunities for organizations to re-visit service delivery models, perhaps offering care in places where young mothers reside (e.g., within maternity residential programs or home visits), or continuing to use telehealth to provide mental health care, a strategy that has increased youth attendance rates at appointments and positively impacted their perceptions of service quality [55].

At the provider level, the young mothers’ own recommendations (Table 6) again closely align with TVIC principles. Fundamentally, young mothers’ physical safety in care settings will be enhanced with greater attention to promoting privacy and confidentially. This includes recognizing that there may be topics that the mother does not wish to discuss in the presence of individuals attending the appointment with her. Prioritizing their emotional safety then requires providers to be respectful, actively listen, and be present during the encounter, and focus on inquiring about and first addressing the client’s priority concern. Using narrative approaches, in comparison to more structured approaches, to history taking and assessment further reduces the rigid feel of many appointments and increases flexibility by allowing the client to control the narrative and share what is most meaningful to them about their health and experiences [56]. It is important for this population to not be given advice or lectured to, rather they prefer to be given choices and control or involvement in decision-making. SmithBattle et al. [5] encourages providers to “engage teen mothers as experts on their own lives and not passive recipients of professional expertise” (p. 8). In acknowledging that the social worlds of young mothers differ significantly from the professional worlds of clinicians, it provides opportunities for those providing care to recognize that it is their responsibility to reduce their use of medical jargon and when providing solutions or recommendations for treatment, to provide anticipatory guidance- or to clearly describe to the client what they can expect to happen next [14]. Finally, when clients require additional support or specialized mental health care, it is incumbent for the referring provider to employ a process of warm referrals. Warm referrals are a type of system navigation where the referral source actively supports the client to manage the process of contacting, accessing, and engaging with a new provider or organization [57].

### Strengths and limitations

The overall trustworthiness of this qualitative descriptive study was enhanced through the use of several strategies. Credibility was enhanced through triangulation of survey, interview, and ecomap data, allowing for a more comprehensive description of young mothers’ experiences. The introduction of the ecomap as an elicitation technique in the interview increased the depth of interaction between the researcher and the study participant and allowed for the emergence of richer and more detailed descriptions of their experiences. Data dependability was promoted through the use of code-re-code procedures during analysis. This form of qualitative inquiry does have its limitations. We initially sought to introduce variation into the purposeful sample by recruiting young mothers who self-reported being “high” or “low” users of mental health services in the quantitative study component. However, what emerged during the qualitative interviews was that users’ perceptions or experiences of their mental health care was not influenced by the number of services accessed. Also, a majority of the participants had lived at or accessed wrap-around services from community-based “maternity homes,” which limits the transferability of the findings. Young mothers who have limited social or professional support to address their mental health concerns during these periods may have different experiences or reasons for not accessing care. However, given that the young mothers in this study did have extensive experience working with a range of providers, they were able to reflect thoughtfully on their care experiences and provide a substantive list of attributes that they found to have either facilitated or inhibited their decision to use mental health care.

## Conclusion

This study aimed to deepen our understanding of mental health service use by young mothers. Our participants were young, mostly single mothers with mental health problems. These young mothers parented under complex conditions, often grounded in histories of loss, trauma and ACES, and their transition to parenthood included social and economic disadvantage and stigmatization.

In terms of support, these mothers reported the need for at least one individual who they could talk to about mental health at a time that is convenient for them and when their need was greatest. Throughout the perinatal period however, the majority of participants sought out both social and professional support. While factors at multiple levels influenced their decisions and capacity to access mental health services, young women expressed reservations about using services where they felt judged, stigmatized, or unsafe. This was clear across all levels of interaction with providers and systems. Historical experiences of poor care creates a hesitancy for further engagement and is a huge barrier to safe engagement with systems of support. Fundamentally, health and social care services providing mental health support to young mothers must be transformed to meet the needs of service users. TVIC principles implemented at individual and organizational levels provide key strategies for achieving this goal. We have attempted to capture and present this information in a way to inform services working with these mothers. Increased ability to assist these mothers individually and as parents will positively impact their children and the next generation.

## Data Availability

The data sets generated and analysed during the current study are not publicly available (due to sensitive information from participants, in particular the narratives of their life and health histories contained within the qualitative interviews) but are available from the corresponding author on reasonable request.

## Abbreviations

NFP: Nurse-Family Partnership
TVIC: Trauma-and violence-informed care
